# Impact of low-*x* resummation on QCD analysis of HERA data

**DOI:** 10.1140/epjc/s10052-018-6090-8

**Published:** 2018-08-03

**Authors:** xFitter Developers’ team, Hamed Abdolmaleki, Valerio Bertone, Daniel Britzger, Stefano Camarda, Amanda Cooper-Sarkar, Francesco Giuli, Alexander Glazov, Aleksander Kusina, Agnieszka Luszczak, Fred Olness, Andrey Sapronov, Pavel Shvydkin, Katarzyna Wichmann, Oleksandr Zenaiev, Marco Bonvini

**Affiliations:** 1 Faculty of Physics, Semnan University, Semnan, 35131-19111 Iran; 2 Department of Physics and Astronomy, VU University, 1081 HV Amsterdam, The Netherlands; 3 NIKHEF Theory Group, Science Park 105, 1098 XG Amsterdam, The Netherlands; 4 Physikalisches Institut, Universität Heidelberg, Im Neuenheimer Feld 226, 69120 Heidelberg, Germany; 5 CERN, 1211 Geneva 23, Switzerland; 6 Particle Physics, Denys Wilkinson Bdg, Keble Road, University of Oxford, OX1 3RH Oxford, UK; 7 Deutsches Elektronen-Synchrotron (DESY), Notkestrasse 85, 22607 Hamburg, Germany; 8 Institute of Nuclear Physics, Polish Academy of Sciences, ul. Radzikowskiego 152, 31-342 Cracow, Poland; 9 T. Kosciuszko Cracow University of Technology, 30-067 Cracow, Poland; 10 SMU Physics, Box 0175 , Dallas, TX 75275-0175 USA; 11 Joint Institute for Nuclear Research (JINR), Joliot-Curie 6, 141980 Dubna, Moscow Region Russia; 12 INFN, Sezione di Roma 1, Piazzale Aldo Moro 5, 00185 Rome, Italy

## Abstract

Fits to the final combined HERA deep-inelastic scattering cross-section data within the conventional DGLAP framework of QCD have shown some tension at low *x* and low $Q^2$. A resolution of this tension incorporating $\ln (1/x)$-resummation terms into the HERAPDF fits is investigated using the xFitter program. The kinematic region where this resummation is important is delineated. Such high-energy resummation not only gives a better description of the data, particularly of the longitudinal structure function $F_L$, it also results in a gluon PDF which is steeply rising at low *x* for low scales, $Q^2 \simeq 2.5\,\hbox {GeV}^2$, contrary to the fixed-order NLO and NNLO gluon PDF.

## Introduction

Deep-inelastic-scattering (DIS) experiments have traditionally been used to probe the parton distribution functions (PDFs) of the proton. A very broad range of resolving power, as characterised by $Q^2$ (the negative four-momentum transfer squared) and by Bjorken *x* (which is interpreted as the fraction of the proton’s momentum taken by the struck parton), was accessed at HERA. Perturbative quantum chromo-dynamics (pQCD) is expected to describe these data, such that PDFs can be extracted for $Q^2 \gtrsim 2$–$3\,\hbox {GeV}^2$.

The final combined inclusive cross-section data from the HERA experiments H1 and ZEUS [1] were input to QCD analyses using fixed-order pQCD at LO, NLO and NNLO to provide the HERAPDF2.0 set of parton distributions. However, some tension was observed at low $Q^2$ such that the $\chi ^2$ for these fits drops steadily as the minimum energy $Q_{\mathrm{min}}^2$ of the data entering the fit is raised up to $Q^2_{\mathrm{min}}\simeq 10\,\hbox {GeV}^2$ (see Fig. 19 of Ref. [1]). This turns out to be true for all perturbative orders and is not mitigated by going to higher order. In particular, the $\chi ^2$ of the NNLO fits is not better than the NLO fit for low values of $Q_{\mathrm{min}}^2$.

A further observation is that the increased $\chi ^2$ of the fits to the low-$Q^2$ data is largely attributable to the kinematic region of low *x* and high *y* (where *y* = $Q^2/sx$ and $\sqrt{s}$ is the centre-of-mass energy) in the neutral-current reduced cross-section data $\sigma _{\mathrm{red}}$, defined as^1^:

1 $$\begin{aligned} \sigma _{\mathrm{red}} =F_2 - \frac{y^2}{Y_{+}} F_L, \end{aligned}$$

where, $F_2$ and $F_L$ are the structure functions, which are related to the parton distributions [2], and $Y_{+}= 1 + (1-y)^2$. In this kinematic region (low $Q^2$ and low *x*) the data take a turn-over (see Fig. 7 and e.g. Fig. 59 of Ref. [1]). This effect can be ascribed to the negative term proportional to $F_L$ in Eq. (1). However, fits to data using fixed-order pQCD do not describe this turn-over very well, suggesting that a larger $F_L$ is needed for a better description. This in turn suggests that the gluon evolution may need modification since $F_L$ is closely related to it [3].

It has been noted that the addition of a higher-twist term to the $F_L$ structure function improves the quality of the fits [4–7]. Such a higher-twist term improves the $\chi ^2$ both at NLO and NNLO, so that the NNLO $\chi ^2$ becomes better than the NLO one. Moreover, it also improves the description both of the low-*x*, high-*y* reduced cross sections and the $F_L$ data from HERA [8] (see Figs. 4, 5, and 11 of Ref. [4]).

Recently, an alternative approach which can improve the description of low-$Q^2$ data has been proposed. Since the kinematics of HERA is such that low-$Q^2$ data is also at low *x*, it has been suggested that the DGLAP resummation of $\ln Q^2$ terms should be augmented by $\ln (1/x)$ (BFKL) resummation [9]. This idea is not new: the necessary calculations have been explored in Refs. [10–27]. They also inspired various phenomenological fits, e.g. Ref. [28]. However, a complete implementation, such that these terms can readily be used for fitting PDFs, is new. This was possible thanks to new theoretical developments and the publication of the HELL code which implements $\ln (1/x)$ resummation [29, 30]. The present paper explores the implementation of the public HELL 2.0 code into xFitter [31–33] and the consequences for a HERAPDF-style fit using this code. The conclusions of the study of Ref. [9] are confirmed by our analysis. Having interfaced HELL to the public xFitter tool makes $\ln (1/x)$ resummation accessible for any future PDF determination.

## Input data sets

The final combined $e^{\pm }p$ cross-section measurements of H1 and ZEUS [1] cover the kinematic range of $Q^2$ from $0.045\,\hbox {GeV}^2$ to $50000\,\hbox {GeV}^2$ and of Bjorken *x* from 0.65 down to $6\cdot 10^{-7}$. There are 169 correlated sources of uncertainty and total uncertainties are below $1.5\%$ over the $Q^2$ range $3\,\hbox {GeV}^2< Q^2 <500\,\hbox {GeV}^2$ and below $3\%$ up to $Q^2=3000\,\hbox {GeV}^2$. There are data from neutral-current (NC) and charged-current (CC) processes and for $e^+p$ and $e^-p$ scattering. In addition to this, the NC $e^+p$ data are available for several different proton beam energies. The availability of NC and CC precision data over a large phase space allows for the determination of PDFs. The difference between the NC $e^+p$ and $e^-p$ cross sections at high $Q^2$, together with the high-$Q^2$ CC data, constrains the valence distributions. The lower-$Q^2$ NC data constrain the low-*x* sea quark distributions and their precisely measured $Q^2$ variation constrains the gluon distribution. Furthermore, the inclusion of NC data at different beam energies probes the longitudinal structure function $F_L$ such that the gluon is further constrained. A minimum $Q^2$ cut of $Q^2 \ge 3.5\,\hbox {GeV}^2$ is imposed on inclusive HERA data. This gives 1145 data points included in the fit.

In addition, HERA combined charm [34] and beauty data [35, 36] from ZEUS and H1 are also available. Reduced cross sections for charm production cover the kinematic range $2.5\,\hbox {GeV}^2< Q^2 < 2000\,\hbox {GeV}^2$, $3\cdot 10^{-5}< x < 0.05$. There are 48 correlated sources of uncertainty and the total uncertainties are typically $6\%$ at small *x* and medium $Q^2$ and $10\%$ on average. There are 47 charm data points included in the fit after the $Q^2$ cut of $Q^2 \ge 3.5\,\hbox {GeV}^2$ is imposed. ZEUS reduced cross sections for beauty cover the kinematic range $6.5\,\hbox {GeV}^2< Q^2 < 600\,\hbox {GeV}^2$, $1.5\cdot 10^{-4}< x < 0.035$. There are 13 correlated sources of uncertainty and the total uncertainties range from about $10\%$ to $20\%$. There are 17 ZEUS beauty data points included in the fit. H1 reduced cross sections for beauty cover the kinematic range $5\,\hbox {GeV}^2< Q^2 < 2000\,\hbox {GeV}^2$, $2\cdot 10^{-4}< x < 0.055$. There are 14 correlated sources of uncertainty and the total uncertainties range from about $20\%$ to $40\%$. There are 12 H1 beauty data points included in the fit. The inclusion of charm and beauty data in the fit is useful to determine the optimal charm and beauty pole masses. Additionally, since the charm data in particular extend to rather small values of *x*, they may be sensitive to $\ln (1/x)$ resummation effects.

## Fit strategy

The present QCD analysis uses the xFitter program [31–33] and is based on the HERAPDF2.0 setup. However, in order to facilitate the inclusion of small-*x* resummation, some differences have been introduced with respect to the HERAPDF2.0 theory settings. In this section, we first present our setup, and then highlight the features that differ from those of HERAPDF2.0.

In the present analysis, we use the APFEL code [37] to compute the structure functions and the solution of the DGLAP evolution equations. The APFEL code implements the FONLL variable-flavour-number scheme [38] for the treatment of heavy quarks. The heavy-quark pole masses were initially chosen to be $m_c=1.43$ GeV and $m_b=4.5$ GeV. These choices follow those of the HERAPDF2.0, but the sensitivity to these values is reviewed in Sect. 4. The choice of APFEL is motivated by the fact that it has been interfaced to the HELL code which is needed to include small-*x* resummation. The HELL code is a standalone code that implements the resummation corrections to the DGLAP splitting functions *P* and to the DIS coefficient functions *C* (both massless and massive) up to next-to-leading-log accuracy in $\ln (1/x)$ ($\hbox {NLL}x$).^2^ The output of HELL is in the form of corrections $\varDelta _l P^{\mathrm{N}^k{\mathrm{LL}}x}$ and $\varDelta _l C^{\mathrm{N}^k{\mathrm{LL}}x}$ to the fixed-order quantities $P^{\mathrm{N}^l{\mathrm{LO}}}$ and $C^{\mathrm{N}^l{\mathrm{LO}}}$ (with $k=0,1$ and $l=0,1,2$), which have to be supplied externally. For example, the expressions needed to compute the DGLAP evolution and the DIS structure functions at NNLO+NLL*x* accuracy are given by:

2 $$\begin{aligned} \begin{array}{l} P = P^{\mathrm{N}^2{\mathrm{LO}}} + \varDelta _2 P^{\mathrm{NLL}x},\\ C = C^{\mathrm{N}^2{\mathrm{LO}}} + \varDelta _2 C^{\mathrm{NLL}x}. \end{array} \end{aligned}$$

In our case, the fixed-order contributions are those implemented in APFEL, which is then used in conjunction with HELL to compute DGLAP evolution and structure functions with the inclusion of $\ln (1/x)$ resummation.

QCD evolution yields the PDFs at any value of $Q^2$ if they are parameterised as functions of *x* at some initial scale $Q^2_0$. This scale is chosen to be $Q^2_0= 2.56\,\hbox {GeV}^2$ (i.e. $Q_0=1.6$ GeV). The reason is that the numerical computation of $\ln (1/x)$-resummation corrections may become unreliable at low scales due to the large value of the strong coupling $\alpha _S$. As a consequence, it is safer to keep the initial scale as high as possible: the value $Q_0 = 1.6$ GeV represents a good compromise which is known to lead to reliable results [30]. Also, as all data we are interested in lie above this scale, this choice does not force us to exclude any of the interesting datapoints. However, this choice gives us an initial scale that is above the default charm-quark matching scale $\mu _c$, i.e. the scale at which the number of active flavours switches from $n_f=3$ to $n_f=4$, usually taken to be equal to the charm pole mass $m_c=1.47\,\hbox {GeV}^2<Q_0$. This could appear to be a problem since we wish to fit just the light-quark PDFs and generate the heavy-quark PDFs, including the charm PDF, dynamically. However, since the charm-quark matching scale $\mu _c$ is an unphysical scale, its value can be modified at will, provided it is kept close to $m_c$ to avoid generating large logarithms. Thus, we have used a feature of the APFEL code, discussed in Ref. [39], that allowed us to displace the charm-quark matching scale $\mu _c$ above $Q_0$ while keeping the charm mass fixed at $m_c = 1.43$ GeV. In particular, we have chosen $\mu _c = 1.12\, m_c\simeq 1.6$ GeV, which is slightly larger than $Q_0$.

The quark distributions at the initial scale $Q_0^2$ are represented by the generic form:

3 $$\begin{aligned} xq_i(x,Q_0) = A_i x^{B_i} (1-x)^{C_i} P_i(x), \end{aligned}$$

where $P_i(x)$ defines a polynomial in positive powers of *x*. The parametrised quark distributions $q_i$ are chosen to be the valence quark distributions ($xu_v$, $xd_v$) and the light anti-quark distributions ($x\bar{U}=x\bar{u}$, $x\bar{D}=x\bar{d}+x\bar{s}$). The gluon distribution is parametrised with the more flexible form:

4 $$\begin{aligned} xg(x) = A_g x^{B_g} (1-x)^{C_g}P_g(x) - A'_g x^{B'_g} (1-x)^{C'_g}. \end{aligned}$$

The normalisation parameters $A_{u_v}$ and $A_{d_v}$ are fixed using the quark counting rules and $A_g$ using the momentum sum rule. The normalisation and slope parameters, *A* and *B*, of $\bar{u}$ and $\bar{d}$ are set equal such that $x\bar{u} = x\bar{d}$ at very small *x*. The strange PDFs *xs* and $x\bar{s}$ are parametrised as $xs = x\bar{s}=0.4x\bar{D}$, representing a suppression of strangness with respect to the light down-type sea quarks, but the input data are not sensitive to the fraction of strangeness. Terms with positive powers of *x* are included in the polynomial $P_i(x)$ only if required by the data, following the procedure described in Ref. [32]. This leads to the additional terms $P_{u_v}(x)=1+ E_{u_v} x^2$ and $P_{\bar{U}}= 1 + D_{\bar{U}} x$ and gives a total of 14 free parameters.^3^ The reference value of the strong coupling constant is set to $\alpha _S(M_Z) = 0.118$.

The setting presented so far differs from the one of the HERAPDF2.0 analysis in some respects that we now highlight.

- For the HERAPDF2.0 analysis the DGLAP evolution and the light-quark coefficient functions are taken from the QCDNUM code [40] up to NNLO. There is no difference between the results of QCDNUM and APFEL for the treatment of light quarks. However, APFEL implements the FONLL variable-flavour-number scheme [38], not the TR “optimal” variable-flavour-number scheme of Refs. [41, 42], which is the default in HERAPDF analyses. The choice of the variable-flavour-number scheme represents the first main difference of the present analysis with respect to HERAPDF2.0.
- A second difference is the scale at which PDFs are parameterised. In this analysis we have chosen $Q^2_0= 2.56\,\hbox {GeV}^2$ as compared to 1.9 $\hbox {GeV}^2$ of HERAPDF2.0.
- Furthermore we have chosen the charm-quark matching scale as $\mu _c = 1.12 m_c\simeq 1.6$ GeV, as explained above. This represents the third main difference with respect to the HERAPDF2.0 analysis. Note that, once this new setting is adopted, the optimal values of the charm and beauty masses may change and need to be reassessed (see Sect. 4), thus representing an extra (minor) difference with respect to HERAPDF2.0.

The impact of these differences will be investigated in Sect. 4 before including resummation effects.

**Table 1:** The $\chi ^2$ per degree of freedom (d.o.f.) for PDF fits under different conditions, starting from the settings of the HERAPDF2.0 analysis at NNLO

	Step-1	Step-2	Step-3	Step-4	Step-5
	HERAPDF2.0 NNLO	$\hbox {TR}\rightarrow \hbox {FONLL-C}$	Raise the charm matching scale $\mu _c$	Raise the initial scale $Q_0$	Include $\hbox {NLL}x$ resummation
HERA $\chi ^2/{\mathrm{d.o.f.}}$	1363/1131	1387/1131	1390/1131	1388/1131	1316/1131

**Fig. 1:**
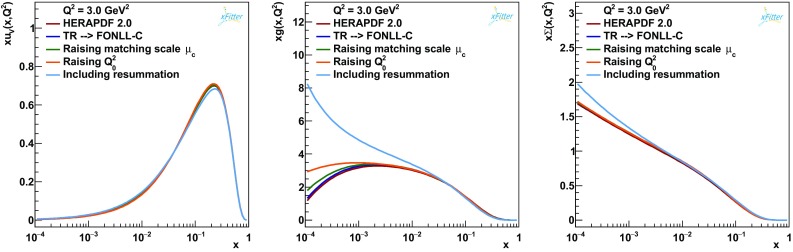
The up valence PDF $xu_v$, the gluon PDF *xg* and the total singlet PDF $x\varSigma$ for each of the 5 steps outlined in the text

It is also useful to compare the present settings with those used in the NNPDF3.1sx analysis [9]. Indeed, the same tools have been used to compute structure functions and PDF evolution, namely APFEL and HELL. The differences, however, are significant. First, the fit methodology is very different, as NNPDF uses a Monte Carlo approach with neural network parametrisation of PDFs. Second, the data considered in NNPDF3.1sx include several additional DIS data sets from other experiments. In addition, that analysis also includes Tevatron and LHC data. Most importantly from the theory point of view, there is a difference in the way charm is treated. In particular, in the NNPDF analysis the charm PDFs are fitted to data. In this analysis, a more conventional approach is used in which the charm PDFs are generated perturbatively. This approach in the framework of the FONLL scheme allows for the inclusion of a damping factor that suppresses subleading higher-order corrections that might be significant at scales comparable to the charm mass [38]. We include this damping factor in our computation as it turns out to improve dramatically the description of the data at NNLO. We will further comment on the effect of this damping factor in Sect. 4.3.

## Results

The effect of $\ln (1/x)$ resummation on splitting functions and DIS coefficient functions is more dramatic at NNLO than at NLO [30]. In fact, the full calculation with NNLO+NLL*x* resummation is closer to the NLO result than it is to the NNLO result. This is not accidental and is mostly due to the perturbative instability of the NNLO correction to the splitting functions generated by small-*x* logarithms [9]. Thus, to better assess the impact of the $\ln (1/x)$ resummation on the original HERAPDF analysis, we only focus on NNLO fits.

**Fig. 2:**
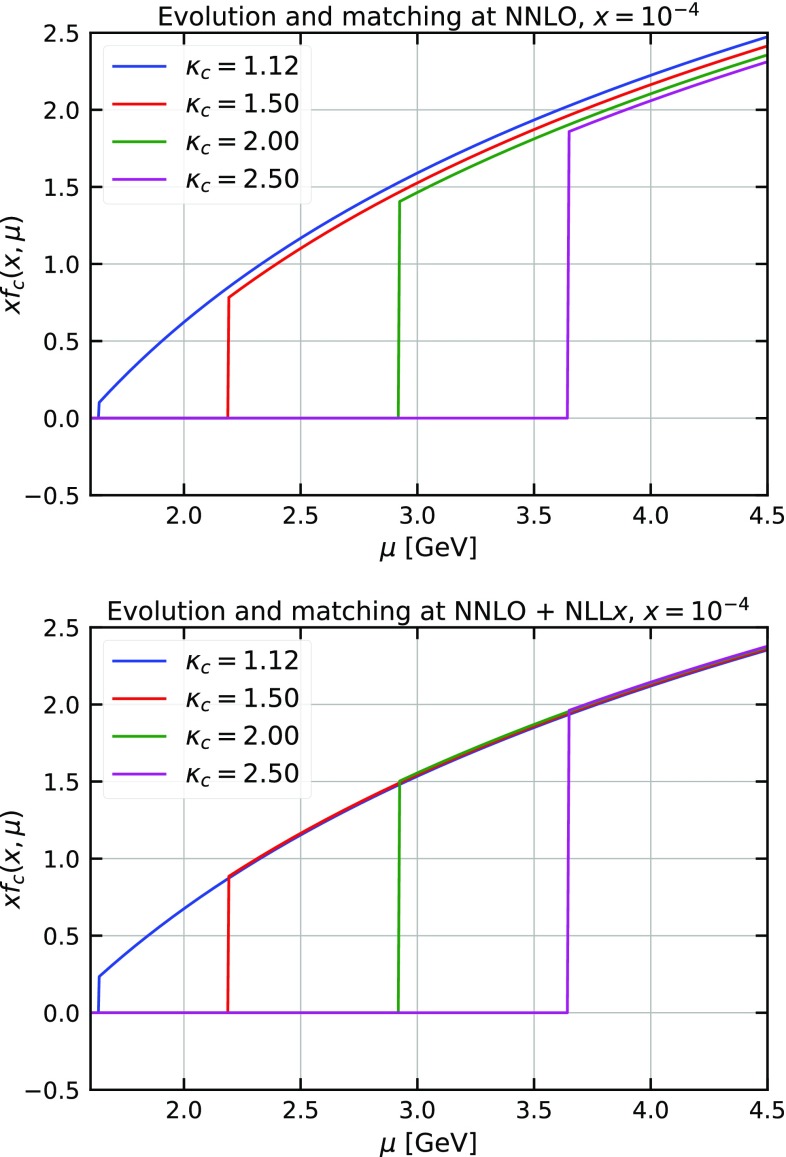
The charm PDF at $x=10^{-4}$ as a function of the factorisation scale $\mu$ for different values of the charm-quark matching scale $\mu _c = \kappa _c m_c$, with $\kappa _c=1.12,1.5,2,2.5$. The plots show the effect of the matching at NNLO (upper plot) and at NNLO+NLL*x* (lower plot)

### *Transition to the new fit settings*

Since the setup described in Sect. 3 differs in various respects from that of the HERAPDF2.0 analysis, we first investigate the effect of these changes in the determination of PDFs. A step-by-step approach is followed. The changes in the fit quality are summarised in Table 1, while the effect on the $u_v$, the total singlet and the gluon PDFs^4^ is shown in Fig. 1.

The starting point is the HERAPDF2.0 analysis, that has a $\chi ^2$ of 1363 units for 1131 degrees of freedom (see Table 4 of Ref. [1]) using only the HERA inclusive data. First we move to the use the FONLL-C scheme [38] in place of the TR scheme [41, 42], with all the same settings. The PDFs look remarkably similar to the HERAPDF2.0 results as illustrated in Fig. 1. However, the $\chi ^2$ increases significantly to 1387. This was expected because FONLL-C is known to lead to a worse description of the data than the TR scheme at this order, as discussed in Ref. [1] (see Fig. 20 of that reference). The origin of this deterioration is related to the details of the construction of the observables within each scheme, which differs in various respects, from the perturbative orders at which each individual contribution is retained to the presence of phenomenological smoothing functions. A full assessment of these differences and their importance for the description of HERA data is beyond the scope of this paper. Some considerations on the impact of the details of the heavy-flavour scheme in our fits with and without $\ln (1/x)$ resummation will be given in Sect. 4.3.

In the next step the charm-quark matching scale $\mu _c$ is moved from $\mu _c=m_c=1.43$ GeV to $\mu _c=1.12m_c\simeq 1.6$ GeV. The $\chi ^2$ remains effectively unchanged, i.e. $\chi ^2=1390$. Again, PDFs do not change significantly, the only exception being the gluon PDF which is slightly enhanced at low values of *x*. The origin of this enhancement can be traced back to the PDF matching conditions. The upper plot of Fig. 2 shows that at low *x* ($x=10^{-4}$) moving up the charm-quark matching scale and using fixed-order NNLO matching conditions has the effect of slightly depressing the charm PDFs at large scales. Since in our fits charm is generated dynamically mostly by gluon splitting, in order to describe the charm component of the experimental data close to the charm-quark matching scale, the gluon PDF must become slightly larger at low *x* to compensate. Interestingly, PDF matching conditions in this region are also affected by large logarithms of 1 / *x*. These logarithms are resummed in HELL exactly like those in the splitting functions and in the DIS coefficient functions. Adding $\ln (1/x)$ resummation to matching conditions and PDF evolution leads to the result in the lower plot of Fig. 2. It is evident that the spread caused by the matching (namely, the residual uncertainty from missing higher orders) is significantly reduced when introducing $\ln (1/x)$-resummation effects. This shows that our results are particularly stable upon displacement of the charm-quark matching scale when resummation is included.

In the subsequent step the initial scale is then safely moved from $Q_0^2=1.9\,\hbox {GeV}^2$ to $Q_0^2=2.56\,\hbox {GeV}^2$. The $\chi ^2$ does not change significantly, i.e. $\chi ^2=1388$. Again, PDFs are mostly unaffected with the exception of the gluon PDF, which is enhanced, mostly at low values of *x*. This change of the PDF shape with $Q_0$ represents a parametrisation uncertainty. Such uncertainties are included in the total PDF uncertainty and thus it will be accounted for in the assessment of the impact of resummation (see Fig. 4 later and discussion thereof).

Finally, in the last step $\ln (1/x)$ resummation at NLL*x* is turned on. The $\chi ^2$ of the fit falls to 1316. At this step the gluon PDF at $Q^2=3\,\hbox {GeV}^2$ differs significantly from that of HERAPDF2.0, and also from that of the intermediate (fixed-order) steps, being much steeper at low *x* (see Fig. 1). The total singlet also changes visibly at small *x*. Non-singlet quark PDFs, instead, are insensitive to $\ln (1/x)$ resummation.

The above procedure clearly illustrates the improvement in $\chi ^2$ deriving from $\ln (1/x)$ resummation, and the impact on the gluon and singlet PDFs. However, before assessing the significance of the resummation by studying PDF uncertainties, a few refinements are still needed.

Firstly, given that we now have a completely different shape of the gluon PDF, it is necessary to investigate if the parametrisation used for HERAPDF2.0 is adequate. A parametrisation scan was performed in the FONLL-C scheme with $\ln (1/x)$ resummation and the parametrisation of HERAPDF2.0 was confirmed. However, the negative term in the gluon (see Eq. (4)) is now small, being compatible with zero within three standard deviations, to be compared to more than five standard deviations for HERAPDF2.0. In fact, this is also the case for the fit without $\ln (1/x)$ resummation and is due to the higher starting scale $Q^2_0=2.56\,\hbox {GeV}^2$. This suggests that the parametrisation uncertainty previously found when raising $Q_0$ is likely to be reduced at this scale. Nevertheless, the shape of the low-*x* gluon is very different for the fits with and without $\ln (1/x)$ resummation, being flattish/decreasing for the standard NNLO fit and steep for the NNLO+NLL*x* fit. We return to this point below.

**Fig. 3:**
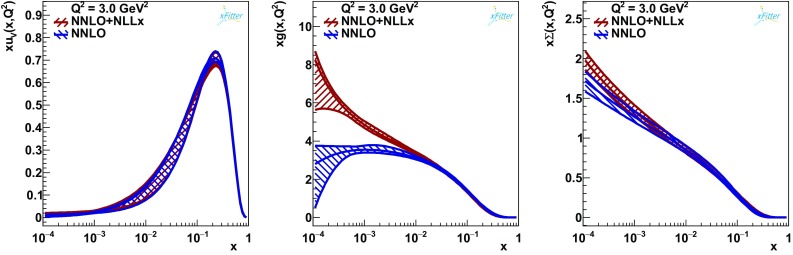
The up valence PDF $xu_v$, the gluon PDF *xg* and the total singlet PDF $x\varSigma$ for the final fits with ($\hbox {NNLO+NLL}x$) and without (NNLO) $\ln (1/x)$ resummation

Secondly, the charm and beauty mass values used may not be optimal for the new settings. Thus charm [34] and beauty data [35, 36] from HERA are included in the fit to perform a charm-mass scan and a beauty-mass scan. Various fits have been performed by changing the charm and beauty pole masses $m_c$ and $m_b$. The optimal values $m_c=1.46$ GeV and $m_b=4.5$ GeV are obtained from the NNLO+NLL*x* fits. The value of the charm-quark matching scale is also moved accordingly to $\mu _c \simeq 1.64$ GeV, to keep $\mu _c/m_c=1.12$. Note that the $\chi ^2$ variation over a range of 0.1 GeV for $m_c$ and 0.3 GeV for $m_b$ reaches 1 unit at most. This insensitivity is similar for both the fixed-order and resummed fits, thus showing a good stability of the fits for the variation of these physical parameters.

Since the charm data are in a kinematic region in which $\ln (1/x)$-resummation corrections are important, this data set will also be included in our final fits. The beauty measurements mostly lie at higher *x* and $Q^2$ and thus are not expected to give a significant contribution in the region of interest. Indeed, we have verified that including these data in the fit does not change the PDFs in any appreciable way. Moreover, the $\chi ^2$ of the beauty datasets computed from PDFs determined with and without those data are basically the same. This is mostly due to the fact that these datasets contain only a very small number of datapoints (29 in total) with large uncertainties. While their inclusion in the fit does not impact the PDF determination, we decided to retain them for our main results.

### *Final results with full uncertainties and comparison with data*

The final fits that we are going to present use HERA inclusive, charm and beauty data with the new values of $m_c=1.46$ GeV, $m_b=4.5$ GeV and $Q^2_0=2.56$ GeV, and make use of the FONLL-C scheme, with and without $\ln (1/x)$ resummation as implemented in HELL. An exploration of various sources of the uncertainties has been performed, following the HERAPDF2.0 prescription. In addition to the experimental uncertainty, which is evaluated using either the Hessian (our default) or the Monte Carlo method, a number of model uncertainties are considered. Specifically, we have varied the charm mass ($\varDelta m_c=\pm 0.05$ GeV), the bottom mass ($\varDelta m_b=\pm 0.25$ GeV), the strong coupling $\alpha _S(m_Z^2)$ ($\varDelta \alpha _S=\pm 0.001$), the fraction of strangeness ($\varDelta f_s=\pm 0.1$), the initial scale ($Q^2_0=2.88\,\hbox {GeV}^2$), and the $Q_{\mathrm{min}}^2$ cut on the data ($Q^2_{\mathrm{min}}= 2.7\,\hbox {GeV}^2$ and $Q^2_{\mathrm{min}}=5\,\hbox {GeV}^2$). Additionally, parametrisation uncertainties have been explored by adding extra terms to the polynomials $P_i(x)$ of Eq. (3). This can give rise to different PDF shapes with only slightly different $\chi ^2$’s from that of the main fit. In the present case, the only noticeable difference comes from the inclusion of a linear term to the polynomial $P_{u_v}(x)$ of the valence up quark PDF (this was also found in the HERAPDF2.0 study). The largest difference on the uncertainty of the gluon distribution arises from the variation of the $Q^2_{\mathrm{min}}$ cut to $5\,\hbox {GeV}^2$. Interestingly, this uncertainty decreases for the fit with $\ln (1/x)$ resummation due to the reduced tensions with the data, see the discussion below.

**Fig. 4:**
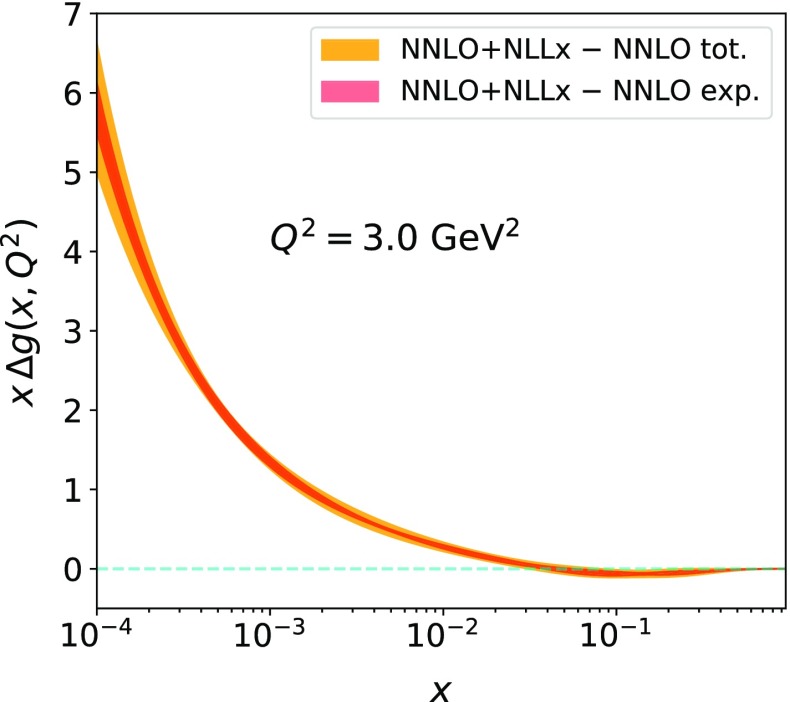
The difference between the gluon distribution determined in the fits at NNLO with and without NLLx resummation taking into account the correlations between their uncertainties. The orange (red) band indicates the full (experimental) uncertainty on the difference

**Fig. 5:**
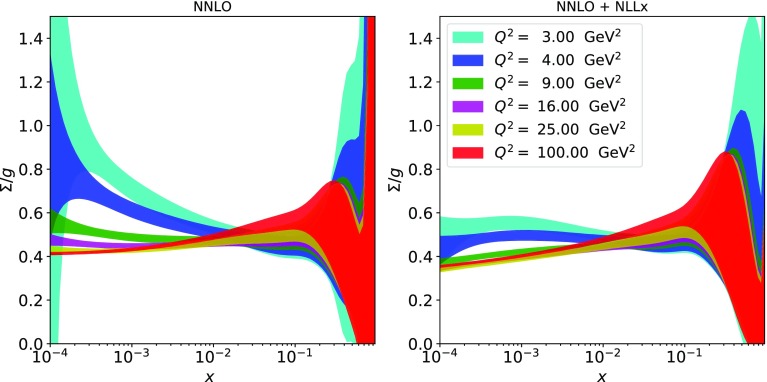
The ratio of the total singlet to the gluon PDF as a function of *x* shown for different scales $Q^2$ for the final fits with (right plot) and without (left plot) $\ln (1/x)$ resummation

Figure 3 shows a direct comparison of PDFs with and without $\ln (1/x)$ resummation at $Q^2 = 3\,\hbox {GeV}^2$. This figure displays also the full uncertainty bands. Note, however, that since the data used in the two fits are the same, the uncertainty bands are highly correlated. In order to quantify the difference in the gluon shape taking into account the correlations, the method developed in Ref. [43] is employed. Specifically, we take a version of our fits with experimental uncertainties estimated using the Monte Carlo method. We then generate replicas of the data using the same random number sequence used for the fits with and without resummation to evaluate the spread of the synchronised differences. A similar procedure is adopted for the model and parameterisation uncertainties (including the uncertainty due to variation of $Q_0$ mentioned above). The difference for the gluon distribution with its uncertainty is shown in Fig. 4. The correlated PDF sets at NNLO and NNLO+NLLx can be used to evaluate the impact of $\ln (1/x)$ resummation on other analyses and can be found on the xFitter public page.

**Fig. 6:**
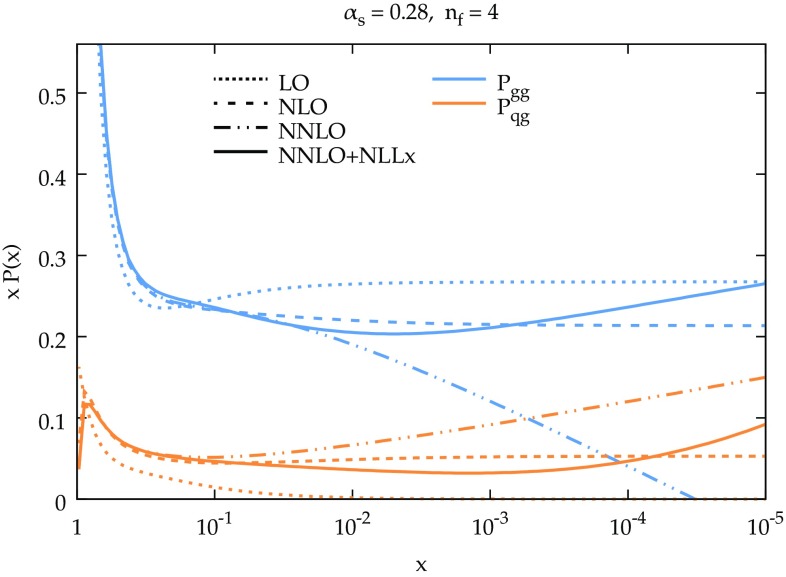
The resummed splitting functions at NNLO+NLL*x* (solid) compared to fixed order at LO (dotted), NLO (dashed) and NNLO (dot-dot-dashed) for $P_{gg}$ (upper curves) and $P_{qg}$ (lower curves) as a function of *x*. The plots are at $\alpha _S(Q^2)=0.28$ (corresponding to $Q^2\sim 4\,\hbox {GeV}^2$) and $n_f=4$

When resummation is included, both the gluon and the total singlet PDFs rise at low *x*. This contrasts with the shape of the gluon when resummation is not included. This behaviour can be studied in more detail by examining the evolution of the ratio $\varSigma /g$ at different scales, as shown in Fig. 5. For the fit without resummation, the ratio exhibits a strong dependence on the scale, ranging from values exceeding unity at low *x* and low scales to values $\sim 0.5$ at high scales. The evolution of the ratio is much more stable when resummation is included, with the total singlet never exceeding the gluon PDF and remaining approximately constant at low *x*. At large scales ($Q\sim 1000$ GeV) and low *x*, the ratio $\varSigma /g$ becomes equal to within a few percent for the fits with and without resummation, while gluon and total singlet remain different at the $50\%$ level. This behaviour of the ratio for the fit without resummation can be explained by a peculiar feature of the $xP_{gg}(x)$ and $xP_{qg}(x)$ splitting functions, see Fig. 6.

For $Q^2\sim 4\,\hbox {GeV}^2$ ($\alpha _S(Q^2) =0.28$), the NNLO splitting function $xP_{gg}(x)$ ($xP_{qg}(x)$) falls (rises) for $x\rightarrow 0$ with $xP_{qg}(x)>xP_{gg}(x)$ for $x \lesssim 10^{-3}$. This causes a suppression of the low-*x* gluon in favour of the total singlet PDF. When resummation is added, the relation $xP_{qg}(x)<xP_{gg}(x)$ is restored down to very low values of *x* leading to a suppression of the total singlet compared to the gluon PDF.

**Table 2:** Total $\chi ^2$ per d.o.f. and some of the partial $\tilde{\chi }^2$’s per number of data points (n.d.p.) for the PDF fits to HERA inclusive and heavy-quark data with and without $\ln (1/x)$ resummation with the new settings. Also shown are the contributions to the $\chi ^2$ from the correlated shifts and the log terms

Total $\chi ^2 (=\tilde{\chi }^2+\text {corr}+\log )/{\mathrm{d.o.f.}}$	NNLO fit with new settings	$\hbox {NNLO+NLL}x$ fit with new settings
	$1468\,(1327+119+22)/1207$	$1394\,(1305+91-2)/1207$
Dataset inclusive $(\tilde{\chi }^2+\text {corr}+\log )/{\mathrm{n.d.p.}}$	$(1264+103+21)/1145$	$(1239+78-4)/1145$
Subset NC 920 $\tilde{\chi }^2/{\mathrm{n.d.p.}}$	447 / 377	413 / 377
Subset NC 820 $\tilde{\chi }^2/{\mathrm{n.d.p.}}$	67 / 70	65 / 70
Dataset charm $(\tilde{\chi }^2+\text {corr}+\log )/{\mathrm{n.d.p.}}$	$(47+12-1)/47$	$(50+11-1)/47$
Dataset beauty $(\tilde{\chi }^2+\text {corr}+\log )/{\mathrm{n.d.p.}}$	$(16+2+3)/29$	$(16+2+3)/29$

The $\chi ^2$ values of the final fits are summarised in Table 2. There is a decrease of 74 units in $\chi ^2$ when $\ln (1/x)$ resummation is used. Most of this difference comes from the highly accurate NC $E_p = 920$ GeV data which probe the low-*x* and low-$Q^2$ region and thus are particularly sensitive to $\ln (1/x)$ resummation. The table also shows the partial $\chi ^2$ for these data, the NC $E_p=820$ GeV^5^ and charm and beauty data, which may also be sensitive. Other data sets entering the fit probe higher *x* and $Q^2$ and their $\chi ^2$ is not significantly affected, and so they are not shown in the table. To fully appreciate the source of the overall improvement in $\chi ^2$, it is necessary to consider the contribution due to the correlated systematic uncertainties and the logarithmic term. The form of the $\chi ^2$ minimised during the fits is given by [44]:

5 $$\begin{aligned} \chi ^2= & {} \displaystyle \sum _i\frac{\left[ D_i -T_i\left( 1-\sum _j\gamma _j^i b_j\right) \right] ^2}{\delta _{i,{\mathrm{unc}}}^2 T_i^2+\delta _{i,{\mathrm{stat}}}^2D_i T_i} + \sum _j b_j^2\nonumber \\&+\displaystyle \sum _i \ln \frac{\delta _{i,{\mathrm{unc}}}^2 T_i^2+\delta _{i,{\mathrm{stat}}}^2D_i T_i}{\delta _{i,{\mathrm{unc}}}^2 D_i^2+\delta _{i,{\mathrm{stat}}}^2D_i^2}, \end{aligned}$$

where $T_i$ is the theoretical prediction and $D_i$ the measured value of the *i*-th data point, $\delta _{i,{\mathrm{stat}}}$, $\delta _{i,{\mathrm{unc}}}$, and $\gamma _j^i$ are the relative statistical, uncorrelated systematic, and correlated systematic uncertainties, and $b_j$ are the nuisance parameters associated to the correlated systematics which are determined during the fit. The “$\tilde{\chi }^2$”, “corr” and “log” contributions reported in Table 2 correspond to the first, second and third terms in the r.h.s. of Eq. (5), respectively. A reduction of the correlated shifts term indicates that the fit does not require the predictions to be shifted so far within the tolerance of the correlated systematic uncertainties, while a reduction of the log term reflects a better agreement of the theoretical predictions with the data. Considering the partial $\tilde{\chi }^2$, the correlated shift term and the log term for the inclusive and heavy-quark data, we can see that the largest improvement comes from the NC $E_p=920$ GeV, which is much better described in the NNLO+NLLx fit. There is no visible improvement in the NC $E_p=820$ GeV data set, perhaps due to the larger uncertainties of its low *x* data. There is no improvement for the beauty data either, and since most of the data points are at higher *x* and $Q^2$ this is not surprising. More surprisingly, the change for the charm data from $\chi ^2=58$ at NNLO to $\chi ^2=60$ at NNLO+NLLx is negligible. This contrasts with the results of Ref. [9]. The origin of this difference is that the FONLL scheme with perturbatively generated charm at NNLO, used in this analysis, provides a better description of the charm data than the FONLL implementation with fitted charm [45, 46] (as also found in Ref. [9]). We will return on this at the end of the section.

**Fig. 7:**
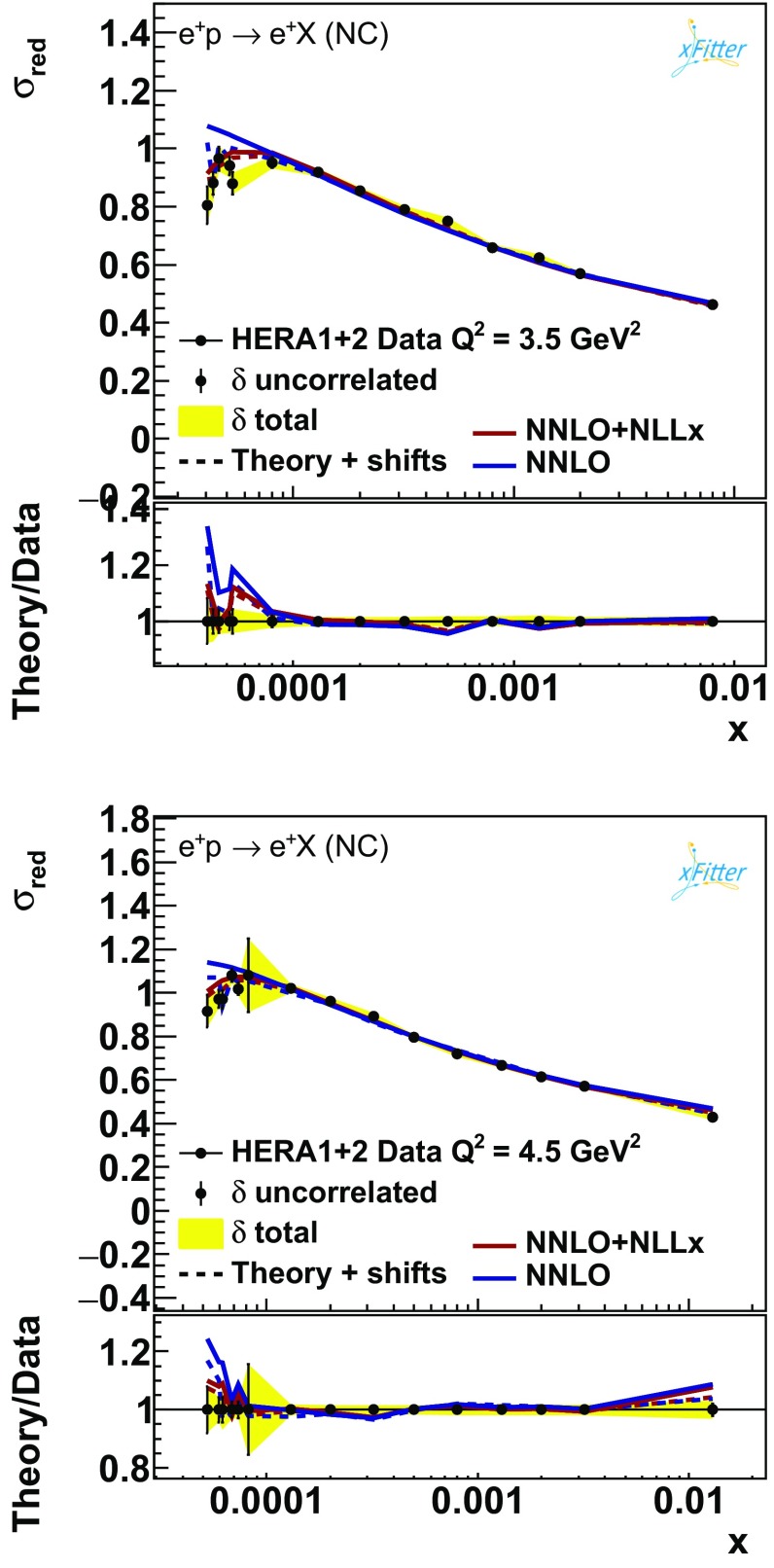
The HERA NC $E_p= 920$ GeV data compared to the fits with and without $\ln (1/x)$ resummation for the $Q^2 = 3.5$ and $4.5\,\hbox {GeV}^2$ bins

In Fig. 7 the results of the fits are compared to the NC $E_p=920$ GeV inclusive reduced cross-section data in the lowest $Q^2$ bins included in the fits. The plots illustrate the predictions both before and after the shifts due to the experimental correlated systematics are applied. The shift to the theoretical prediction $T_i$, according to Eq. (5), is given by $T_i\sum _j\gamma _j^i b_j$. It is evident that for the fit including $\ln (1/x)$-resummation effects the initial description of the data is better and thus the correlated shifts are smaller. In particular, the low-*x* turn-over of the measurements is better reproduced by the fit that includes $\ln (1/x)$ resummation. This is a direct consequence of the steeper gluon at low *x* (see Fig. 3) that makes $F_L$ larger at low *x* causing a more pronounced turn-over of the reduced cross section (*cfr.* Eq. (1)). This is the main reason for the reduction in $\chi ^2$ of the fit with $\ln (1/x)$ resummation.

**Fig. 8:**
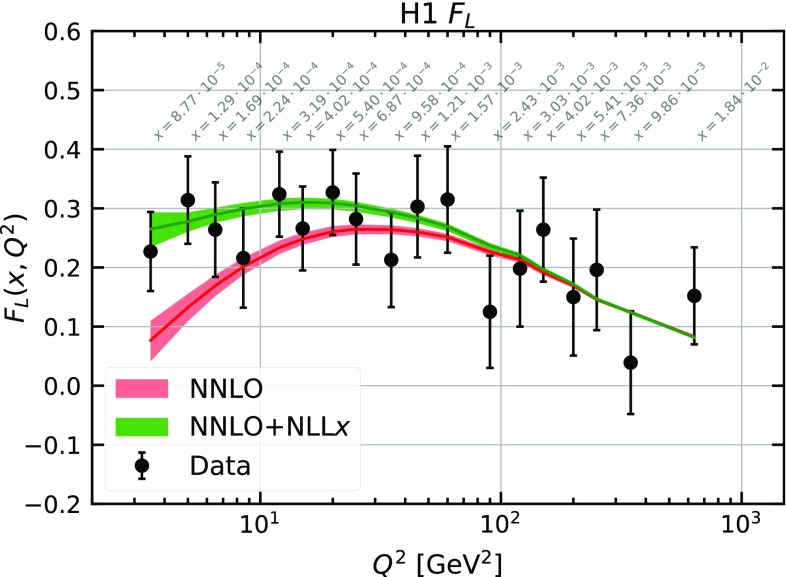
The H1 extraction of $F_L$ compared to the predictions with and without $\ln (1/x)$ resummation

This point is illustrated also in Fig. 8 where the theoretical predictions of $F_L$ with and without $\ln (1/x)$ resummation are compared to the H1 $F_L$ extraction. The visual description of this data set is improved in the former case thanks to the fact that $\ln (1/x)$-resummed predictions for $F_L$ are larger at low *x*.^6^

### *Comparison with the NNPDF analysis*

**Fig. 9:**
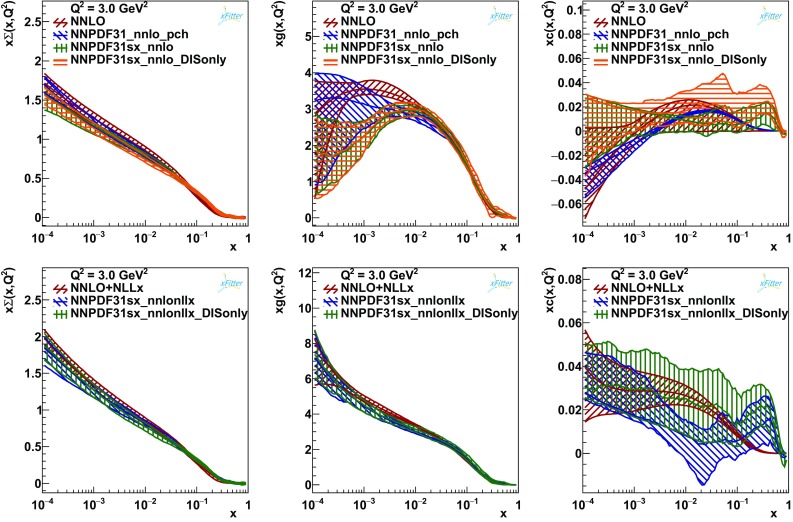
The total singlet, gluon and charm PDFs for the final fits at NNLO (upper plots) and $\hbox {NNLO+NLL}x$ (lower plots) compared to the analogous NNPDF3.1 determinations

We conclude this section by comparing our results with those of the NNPDF3.1 family [9, 47]. In Fig. 9 we show the total singlet, gluon and charm PDFs with (lower plots) and without (upper plots) $\ln (1/x)$ resummation. In particular, on top of our PDFs, we consider the global and the DIS-only PDF sets of Ref. [9] (NNPDF3.1sx, henceforth). In contrast with this analysis, all NNPDF3.1sx sets have been obtained by fitting the charm PDFs to data. Therefore, in order to gauge the impact of the different treatments of the charm PDFs, in the comparisons at fixed order we also consider the NNPDF3.1 set at NNLO of Ref. [47] obtained using perturbative charm.

At fixed order (upper plots of Fig. 9), the quark-singlet PDF (left plot) appears to be very similar for all four PDF sets considered. The gluon PDF (central plot), instead, presents larger discrepancies. In particular, the NNPDF3.1sx distributions (both global and DIS-only) are somewhat different from the gluon obtained in this analysis at small *x*. Given the consistency of the NNPDF3.1sx results, this appears to be the consequence of the different treatment of the charm PDFs rather than the different data sets. The gluon PDF of the NNPDF3.1 set with perturbative charm is closer to our result at low-*x* ($x \lesssim 0.001$) than to the NNPDF3.1sx curves. We also observe that the suppression on the gluon PDF of the NNPDF3.1sx sets causes an enhancement of the charm distribution at small *x* as compared to both this analysis and NNPDF3.1 with perturbative charm (right plot).

Moving to the PDFs with $\ln (1/x)$ resummation (lower plots of Fig. 9), we observe a better agreement between all PDF sets considered. Noticeably, the gluon distributions are now compatible despite the different treatment of the charm. As a consequence, also the charm PDFs at small *x* are in much better agreement. Note also that the uncertainty bands obtained in this analysis are comparable to those of the NNPDF sets, except for the charm PDF at large *x* whose band is larger for the NNPDF3.1sx sets due to the fact that the charm PDFs are fitted to data.

Another striking difference with respect to our analysis is that a significant reduction (by more than 50 units for 47 datapoints) of the $\chi ^2$ of charm production data when including $\ln (1/x)$ resummation was found in Ref. [9]. The origin of such a huge effect can be traced back to the poor quality of the description of charm data at fixed NNLO in the NNPDF3.1sx study. Indeed, the NNPDF3.1sx $\chi ^2$ of this dataset when resummation is included is very similar to that of the present study, differing by just 2 units. The reason of this difference in the quality of the description of charm data at fixed order is related to the treatment of the charm PDFs. However, the discrepancy cannot be ascribed to the fact that the charm PDFs are fitted in Ref. [9]. In fact, fitting the charm PDFs should give more flexibility to better describe the data. Rather, it is the actual construction of the FONLL-C prediction which differs when the charm PDFs are fitted. Specifically, when fitting the charm PDFs, it has been pointed out that an extra contribution, denoted by $\varDelta _\mathrm{IC}$, should be added to the FONLL formula to account for potential intrinsic-charm-initiated contributions [45, 46].

The introduction of this extra term has the consequence that the phenomenological damping factor usually introduced in the FONLL scheme with perturbative charm to suppress subleading higher-order terms in the vicinity of the charm threshold [38], becomes ineffective. Indeed, when the charm PDFs are fitted, and thus a non-perturbative (or intrinsic) component is allowed, the contributions multiplied by the damping are no longer subleading, and cannot therefore be suppressed. The bad description of the charm data at fixed order in Ref. [9] is thus the consequence of three concurring effects: (1) the absence of damping, (2) the presence of the extra contribution $\varDelta _\mathrm{IC}$ to the FONLL formula, and (3) the fitted charm PDFs which makes this $\varDelta _\mathrm{IC}$ contribution sizeable. Since our charm PDFs are generated perturbatively, the $\varDelta _\mathrm{IC}$ contribution is subleading and does not affect our results significantly. Specifically, the effect of adding such $\varDelta _\mathrm{IC}$ term would effectively be equivalent to removing the damping factor. We have thus performed a fixed-order fit without the damping in the FONLL formula and found that, as expected, the results are not significantly affected (in particular, the $\chi ^2$ of the charm dataset remains unchanged). We have also recomputed the $\chi ^2$ of the charm dataset using FONLL without damping and the NNLO PDFs of Ref. [9], which contain fitted charm, and found indeed that the description of the data is worsened, even though not at the level of the results of Ref. [9] (which additionally include the extra $\varDelta _\mathrm{IC}$ contribution to FONLL). Note that the deterioration of $\chi ^2$ in this case comes mostly from the correlated contribution to the $\chi ^2$, second term in Eq. (5). We have also performed the same exercise activating the damping, which effectively suppresses all contributions due to the fitted-charm PDFs in the vicinity of the charm threshold making the result closer to what one obtains in the perturbative charm case. By doing so we find that the description improves significantly, bringing it at the level of our results. Note that similar tests have been performed in the NNPDF3.1sx study (see the discussion in Sect. 4.1 of Ref. [9]), finding compatible results.

The conclusion is that the treatment of charm in the vicinity of charm threshold deserves a very careful analysis, as it depends on many details, which is however beyond the scope of this paper. What is instead relevant for us and very important to notice is that when $\ln (1/x)$ resummation is included the quality of the description of the data is largely independent of the possible differences in the construction of the charm cross section, as noticed also in Ref. [9]: this is another achievement of high-energy resummation.

## The role of low-*x* and low-$Q^2$ data

**Fig. 10:**
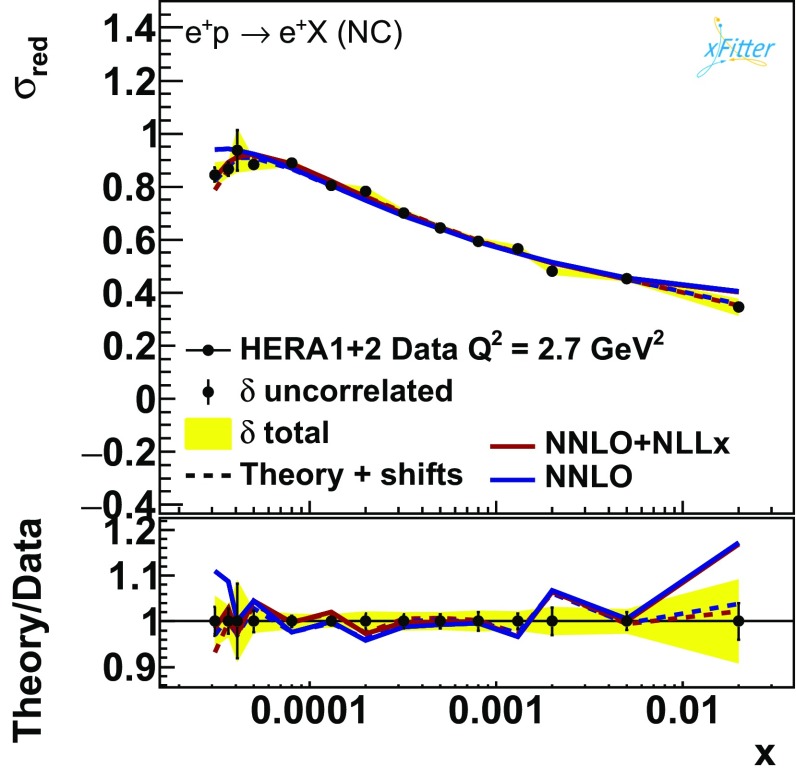
The HERA NC $E_p= 920$ GeV data at $Q^2 = 2.7\,\hbox {GeV}^2$ compared to the fits with and without $\ln (1/x)$ resummation including this bin

So far, we have maintained the restriction of the HERAPDF2.0 analysis, keeping data with $Q^2\ge Q^2_{\mathrm{min}} = 3.5\,\hbox {GeV}^2$. Since the low-$Q^2$ data seem to be better described in the presence of resummation, we can extend the fit down to lower values of $Q^2$ to include the $Q^2=2.7\,\hbox {GeV}^2$ bin of the $E_p=920$ GeV data set,^7^ as was also done in Ref. [9]. A visual inspection of Fig. 10 shows that in the low-*x* region the fit with $\ln (1/x)$ resummation is able to describe these data points better than the fixed-order fit. However, some discrepancies remain at large *x* that are not accommodated by resummation. The PDFs derived from the fits including this extra $Q^2$ bin are very similar to those shown in Fig. 3 and are used to assess the model uncertainty deriving from change in $Q^2_{\mathrm{min}}$ (however, note that the upward variation to $Q^2_{\mathrm{min}}=5$ GeV has a significantly larger impact on the shape of PDFs). We will quantify the goodness of the fits including this low-$Q^2$ bin below.

**Fig. 11:**
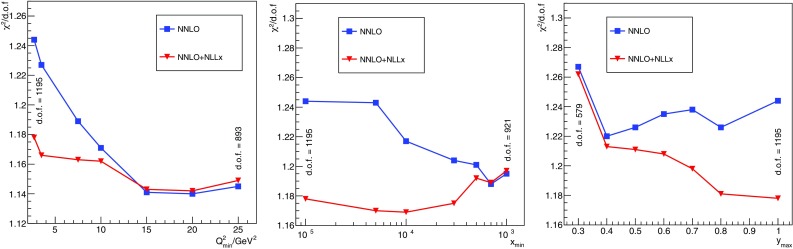
The $\chi ^2$/d.o.f. as a function of $Q^2_{\mathrm{min}}$ (left), $x_{\mathrm{min}}$ (center), and $y_{\mathrm{max}}$ (right). Each plot reports also the number of degrees of freedom at the extremities of each profile

The results presented so far suggest that the improvement of the description of the HERA data when including $\ln (1/x)$ resummation is driven by the low-*x* and low-$Q^2$ data. However, we can delineate the kinematic region responsible for the improvement more precisely. To do so, we have performed $\chi ^2$ scans in $Q^2_{\mathrm{min}}$ with no cut in *x*, and in $x_{\mathrm{min}}$ (where $x_{\mathrm{min}}$ is the minumum value of Bjorken *x* allowed in the fit) fixing $Q^2_{\mathrm{min}} = 2.7\,\hbox {GeV}^2$.^8^ The results are shown in Fig. 11 in the form of $\chi ^2$/d.o.f. profiles. From the $Q^2_{\mathrm{min}}$ scan (left plot) we observe that $\ln (1/x)$ resummation provides a better description of the HERA data from $Q^2_{\mathrm{min}} = 2.7\,\hbox {GeV}^2$ up to $Q^2_{\mathrm{min}}\simeq 15\,\hbox {GeV}^2$, where resummed and fixed-order fits converge towards the same $\chi ^2$ values. The $x_{\mathrm{min}}$ scan (central plot) shows that $\ln (1/x)$ resummation is significantly better than fixed order up to $x_{\mathrm{min}}\simeq 5\cdot 10^{-4}$. This allows us to conclude that $\ln (1/x)$-resummation effects improve the description of the HERA data in the region $x\lesssim 5\cdot 10^{-4}$ and $Q^2\lesssim 15\,\hbox {GeV}^2$.

As mentioned above, a significant part of the improvement observed in the low-$Q^2$ and low-*x* region comes from an improved description of $F_L$. Since $F_L$ contributes to the reduced cross section through a factor $-y^2/Y_+$ (see Eq. (1)), it is instructive to do an additional $\chi ^2$ scan in $y_{\mathrm{max}}$, excluding from the fit data with $y > y_{\mathrm{max}}$. The $\chi ^2$/d.o.f. as a function of $y_{\mathrm{max}}$ is shown in the right plot of Fig. 11. Note that in this scan we set $Q^2_{\mathrm{min}} = 2.7\,\hbox {GeV}^2$ while no cut on $x_{\mathrm{min}}$ is imposed. As expected, the $\chi ^2$ profiles are very similar for small values of $y_{\mathrm{max}}$ while they start diverging for $y_{\mathrm{max}}\gtrsim 0.4$.

In the $\chi ^2$ scans discussed above, full PDF fits were performed for each different cut. In addition to this, we have performed studies in which the $\chi ^2$ are simply re-evaluated for the same value of $x_{\mathrm{min}}$, $Q^2_{\mathrm{max}}$ and $y_{\mathrm{max}}$ using fixed PDFs, specifically those for the NNLO and NNLO+NLL*x* fits with $Q_{\mathrm{min}}^2 = 2.7\,\hbox {GeV}^2$ and no additional cuts in *x* and *y*. Similar trends as those found when refitting were observed. Finally, we have performed a $\chi ^2$ scan and a $\chi ^2$ re-evaluation using the variable $H = \ln (1/x) / \ln (Q^2/ \varLambda ^2)$ with $\varLambda = 88$ MeV defined in Ref. [9]. We find that the low-*H* region is described similarly well by both NNLO and NNLO+NLL*x* fits while at high *H* the $\ln (1/x)$ resummation improves the data description. This is in agreement with the findings of Ref. [9].

**Fig. 12:**
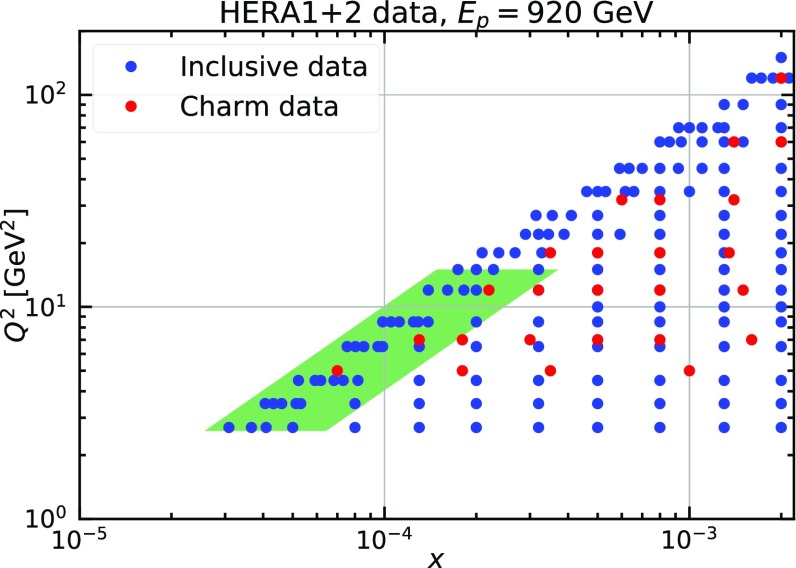
Scatter plot of the low-*x* and low-$Q^2$ kinematic region covered by the HERA1+2 inclusive data and charm data at $E_p= 920$ GeV. The green shaded area indicates the region in which $\ln (1/x)$ resummation has a significant effect as compared to fixed order

The $\chi ^2$ scans as a function of $Q_{\mathrm{min}}$, $x_{\mathrm{min}}$ and $y_{\mathrm{max}}$ allow us to delineate the region of the $(x,Q^2)$-plane in which $\ln (1/x)$ resummation is important.^9^ Figure 12 displays a zoom of the low-*x* and low-$Q^2$ kinematic region covered by the HERA1+2 inclusive and charm data at $E_p= 920$ GeV. The green shaded area indicates the region such that $x<5\cdot 10^{-4}$, 2.7 $\hbox {GeV}^2< Q^2 < 15\,\hbox {GeV}^2$, and $0.4< y < 1$ (assuming $\sqrt{s} = 318$ GeV) determined by combining the results of the scans discussed above.^10^ This provides an estimate of the region where $\ln (1/x)$ resummation provides a significantly better description of the HERA data as compared to fixed order. Since the $\chi ^2$ scans in Fig. 12 have been obtained independently from one another, one may wonder whether the estimate is fully reliable. In order to check this, we have performed two additional fits, one with and one without resummation, excluding only the data points for which $Q^2<15\,\hbox {GeV}^2$ and $y>0.4$. The total $\chi ^2$’s of these fits differ by around 15 units in favour of the resummed fit, mostly due to the correlated and logarithmic terms, to be compared to the 73 units of difference (see Table 2) when the shaded area is included. This confirms that, in the context of DIS, the shaded area in Fig. 12 does provide a reliable estimate of the kinematic region in which resummation works significantly better than fixed order.

## Discussion and summary

The recent implementation of the $\ln (1/x)$-resummation corrections to the DGLAP splitting functions and the DIS coefficient functions in the public code HELL [29, 30] has made possible the determination of PDFs including these effects. This possibility has already been exploited in the recent global analysis of Ref. [9]. In this paper we focused on the study of $\ln (1/x)$-resummation effects on the description of the HERA data in the framework of an HERAPDF analysis. Specifically, we carried out a PDF extraction from the HERA1+2 combined inclusive and charm data [1, 34] in the FONLL-C variable-flavour-number scheme, accurate to NNLO in QCD, including and excluding resummation corrections up to $\hbox {NLL}x$ accuracy. This was possible thanks to the xFitter program [31] interfaced to the APFEL code [37].

The inclusion of the $\ln (1/x)$-resummation effects makes the shape of the gluon PDF at low *x* and low scales steeply rising as opposed to flattish/decreasing of the fixed-order fit (see Fig. 3). The behaviour of the total singlet and gluon PDFs towards low *x* is much more similar when $\ln (1/x)$ resummation is included and the ratio $\varSigma /g$ does not exceed unity in the region of validity of the fit, $Q^2 > 2.56\,\hbox {GeV}^2$. These features make PDFs with $\ln (1/x)$ resummation much more suitable for use in MC generators, such as Sherpa [48], which require positivity of the gluon distribution at all scales, than the standard fixed-order NLO and NNLO PDFs, which have a suppressed gluon PDF at low *x* (however, for consistency, one should also include resummation in the MC generators themselves).

The quality of the fit with $\ln (1/x)$ resummation is significantly better than that of the corresponding fixed-order analysis, indicating a better description of the HERA data. A substantial part of the improvement in the description is driven by $F_L$ which determines the behaviour of the DIS reduced cross section at large values of *y* (*cfr.* Eq. (1)). The improvement is particularly significant at small values of *x* and $Q^2$ due to the relative size of the gluon PDF. In this region the enhancement of $F_L$ caused by resummation helps reproduce the turn-over of the data. The region of the $(x,Q^2)$-plane where resummed predictions provide a better description of the HERA data was delineated in Fig. 12.

In conclusion, $\ln (1/x)$ resummation provides a substantial improvement in the description of the precise HERA1+2 combined data. It represents an alternative to the addition of higher-twist terms [4–7] and does not suffer from the pathological features of some of these analyses [4]. In addition, it overcomes a major disadvantage of the fixed-order analyses, namely a decreasing gluon PDF at low *x* and $Q^2$.

## Footnotes

1 At low $Q^2$ and low *x* the parity-violating term proportional to $xF_3$ can be neglected.

2 The resummation procedure of the logarithmically enhanced terms of the massive coefficient functions implies the computation of the corrections due to $\ln (1/x)$ resummation on the PDF matching conditions. Details can be found in Ref. [30]. These effects are also included in the results presented below.

3 The exact value of $C_g'$ does not matter provided it is large enough that the negative term in Eq. (4) does not contribute at large *x*. We take $C_g' = 25$ following the HERAPDF strategy [1].

4 The $d_v$ is not shown since its shape is unchanged, just as for the $u_v$.

5 In the HERAPDF2.0 combination, the data at $E_p=820$ GeV and $y<0.35$ are combined with $E_p=920$ GeV and attributed to the $E_p=920$ GeV data set. Thus $E_p=820$ GeV data contain only the high $y>0.35$ region which enhances sensitivity to the $\ln (1/x)$ resummation effects.

6 We recall that these data are *not* explicitly included in our fit, but information on $F_L$ is included in the reduced cross sections which are fitted.

7 Due to the limitation of HELL at low scales, we cannot push the $Q^2_{\mathrm{min}}$ cut further down.

8 For these scans the beauty data are not included in the fits, since they have no impact as discussed in Sect. 4.

9 The actual plane over which the constraint acts is the $(x,Q^2/s)$-plane. However, for simplicity, in the following we will only consider the $E_p=920$ GeV inclusive and the charm data sets that were both taken at $\sqrt{s}=318$ GeV.

10 In fact, given the ranges in $Q^2$ and *y*, the constraint on *x* has no effect on the shaded area.
